# Influenza Sentinel Surveillance and Severe Acute Respiratory Infection in a Reference Hospital in Southern Brazil

**DOI:** 10.1590/0037-8682-0498-2017

**Published:** 2019-12-20

**Authors:** Heloisa Zimmerman Faggion, Jaqueline Leotte, Hygor Trombetta, Luciane Aparecida Pereira, Bruna Amaral Lapinski, Meri Bordignon Nogueira, Luine Rosele Vidal, Bernardo Machado Almeida, Ricardo Rasmussen Petterle, Sonia Mara Raboni

**Affiliations:** 1 Universidade Federal do Paraná, Serviço de Doenças Infecciosas, Curitiba, PR, Brasil.; 2 Universidade Federal do Paraná, Laboratório de Virologia, Curitiba, PR, Brasil.; 3 Universidade Federal do Paraná, Serviço de Epidemiologia Hospitalar, Curitiba, PR, Brasil.; 4 Universidade Federal do Paraná, Setor de Ciências da Saúde, Estatística, Curitiba, PR, Brasil.

**Keywords:** Influenza vírus, Seasonal incidence, Viral respiratory infection

## Abstract

**INTRODUCTION:**

We report the results of the active surveillance of influenza infections in hospitalized patients and the evaluation of the seasonality and correlation with temperature and rainfall data.

**METHODS:**

During the 2-year study period, 775 patients were tested for 15 respiratory viruses (RVs).

**RESULTS:**

Most of the 57% of (n=444) virus-positive samples were human rhinovirus and respiratory syncytial virus. However, 10.4% (n=46) were influenza virus (80% FluA; 20% FluB). Age and SARI were significantly associated with influenza. FluB circulation was higher is 2013.

**CONCLUSIONS:**

In the post-epidemic period, influenza remains an important cause of hospitalization in SARI patients.

Influenza viruses cause seasonal infections and are associated with morbidity and mortality. The recommended treatment for influenza is neuraminidase inhibitors, and vaccination might be the best choice for public health prevention, considering the complications among high-risk patients^1^ ^,^ ^2^. The peak timing of influenza-like illness (ILI) varies seasonally, and the characterization of the regional dispersion of this virus are important for introducing preventive measures.

The many influenza A subtypes are determined by the presence of specific hemagglutinin and neuraminidase, and some subtypes are associated with pandemic episodes^1^. Influenza B is classified into two genetic and antigenically distinct lineages, Victoria and Yamagata^1^. Although not a potential pandemic virus, influenza B is often the cause of outbreaks in humans and is characterized by a clinical pattern of intermediate severity. However, fatalities among influenza B cases can occur, especially in the pediatric population^3^. Influenza A subtypes H1N1pdm09 and H3N2 and both influenza B lineages have been detected in Brazil during influenza seasons^4^.

The clinical manifestations of influenza virus infection include fever, headache, myalgia, cough, and sore throat. The signs of severity include oxygen saturation <95%, dyspnea, and respiratory discomfort. The presence of severe disease sets the diagnosis of severe acute respiratory illness (SARI), which also requires notification in the Brazilian health system^5^.

Emerging influenza viruses include A/H7N9, A/H3N2(v), and A/H5N1 subtypes^6^. Surveillance for these emerging viruses is important and includes both active and passive surveillance systems. The goal of these systems is the rapid and early identification of potentially epidemic strains to prevent their spread. Surveillance systems are designed to detect triggers with possible hazards to public health to provide early warning and establish measures to minimize the risks of global effects^7^. 

This study reported the findings of 2 consecutive years of influenza infection active surveillance and respiratory virus (RV) investigation conducted in hospitalized patients, evaluated influenza seasonality in this region, correlated virus circulation with temperature and rainfall data, analyzed the epidemiological and clinical impacts, and compared the severity of influenza infections to those of other respiratory virus (ORV) infections.

This cross-sectional study was approved by the Institutional Ethics Review Board and performed at Hospital de Clínicas/Universidade Federal do Paraná (HC/UFPR), Southern Brazil.

Overall, 775 patients were selected from two databases. (i) The first group included 321 hospitalized individuals who underwent laboratory testing for RVs in 2012 (127/321; 40%) and 2013 (194/321; 60%). Medical charts of the selected group were reviewed, focusing on epidemiology, clinical manifestations, outcomes, laboratory findings, and SARI diagnosis criteria. (ii) The second group included another 454 cases of hospitalized patients in HC/UFPR that were previously notified as SARI by the Epidemiology Hospital staff during 2012 and 2013. Epidemiological and clinical data for this group were electronically retrieved from the SARI notification system. SARI was defined as influenza-like illness along with signs of severity (dyspnea, oxygen saturation <95%, or respiratory discomfort)^5^.

Meteorological data for Curitiba-Paraná (Brazil), including monthly average temperature (°C) and rainfall (mm^3^) from January 2012 to December 2013, were obtained from the Sistema Meteorológico do Paraná (SIMEPAR) database. Curitiba is located in southern Brazil, at latitude 25.5°S, and has a temperate climate.

RVs were detected by multiplex reverse transcription polymerase chain reaction (RT-PCR) using the Seeplex® RV15 ACE Detection kit (Seegene Inc, Korea, according to the manufacturer’s protocol. This kit enables the simultaneous detection of multiple RVs: human adenovirus, human metapneumovirus, parainfluenza viruses (PIV-1, PIV-2, PIV-3, and PIV-4), influenza A (FLUA), influenza B (FLUB), respiratory syncytial virus (RSV-A, RSV-B), human rhinovirus type A and B (HRV A/B), human enterovirus, and human bocavirus, and human coronavirus type 229E/NL63 (alfa-coronaviruses) and OC43/HKU1 (beta-coronaviruses). Influenza A subtyping was performed using real-time RT-PCR (rtRT-PCR) according to the Centers for Disease Control and Prevention protocol^8^. Influenza B-positive samples were tested to identify lineages using a single one-step rtRT-PCR, as previously reported^9^.

Data were analyzed using R (R CORE TEAM, 2018), version 3.3.4. Parametric and non-parametric tests were used to assess differences between continuous variables with normal and asymmetric distributions, respectively. Fisher’s exact and Pearson’s chi-square tests were used to assess differences between categorical variables, as appropriate. Covariates were examined in univariate analysis to determine their association with influenza and other RV infections. Those with *p*<0.05 were included in the multivariate analysis. Using a stepwise conditional procedure, multivariate logistic regression models were conducted to identify independent predictors considering the presence of influenza or other RV infection as the endpoint. To verify the quality of the fit (goodness-of-fit), we used the Hosmer-Lemeshow test and qq-plot graph; both showed a good fit of the proposed model to the data. All *p*-values were two-tailed and *p*<0.05 was considered statistically significant.

Overall, 444 (58.8%) patients had virus-positive samples. As seen in Table 1, influenza virus was detected in 46 samples (10.4%), including 37 (80%) with influenza A and nine (20%) with influenza B. The most commonly detected virus was HRV (162 cases). Viral co-detection occurred in 127 cases (29% of positive samples); among the influenza virus infections, we found eight co-detections, mainly with HRV. Compared to ORVs, there was a lower rate of co-detection among influenza cases (17%). Whether viral co-infections may enhance pathogenicity during infection requires further investigation.

**TABLE 1:** Viruses detected in 444 positive respiratory samples at a tertiary hospital in southern Brazil, 2012 and 2013.

Virus	Single virus	Viral co-detection	Total (%)
	n (%)	n (%)	
HRV a/b	74 (45.7)	88 (54.3)	162 (36.4)
RSV	103 (64)	58 (36)	161 (36.3)
HEV	15 (25)	45 (75)	60 (13.5)
FLU	38 (82.6)	8 (17.4)	46 (10.4)
PIV	18 (50)	18 (50)	36 (8.1)
ADV	11 (30.6)	25 (69.4)	36 (8.1)
HMPV	25 (69.4)	11 (30.6)	36 (8.1)
HCOV	25 (73.5)	9 (26.5)	34 (7.7)
HBOV	7 (29)	17 (71)	24 (5.4)
Total	317	127	444

**HRV:** human rhinovirus; **RSV:** respiratory syncytial virus; **HEV:** human enterovirus; **FLU:** influenza virus; **PIV:** parainfluenza virus; **ADV:** human adenovirus; **HMPV:** human metapneumovirus; **HCOV:** human coronavirus; **HBOV:** human bocavirus.

Overall, 83% (31/37) of influenza A samples were subtyped; of these, 24 (65%) were A/H1N1pdm09 and seven (20%) were seasonal A/H3. Lineage differentiation of the influenza B-positive revealed that all were Victoria-like (Figure 1).

**FIGURE 1:**
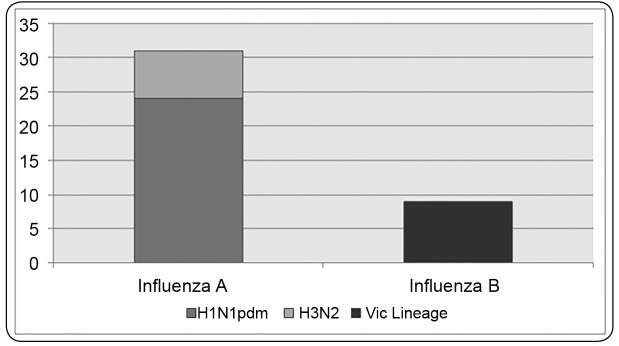
Influenza A subtypes and influenza B lineage characterization.

Both influenza and ORV were predominant in children aged <2 years. This represented 35% (16/46) of the influenza cases and 73% (290/398) of ORV cases. The median age differed significantly between influenza and ORV cases, at 5.4 and 0.7 years, respectively (Table 1). Influenza infection affected more patients aged 14-60 years, a population that remains susceptible to the virus, as the public health system provides the vaccine to children (<5 years), elderly (>60 years), health workers, and patients with comorbidities. In addition, the likelihood of influenza infection increased proportionally with age.

The vaccination status data had a large amount of missing information due to the retrospective nature of this study. Of 46 patients with influenza, only 26% had records of receiving the influenza vaccine in the last year. These findings emphasize the need to expand immunization coverage in high-risk patients. Most patients infected had not received the vaccine or those who presented a low immune response^10^.

Unlike in immediate post-pandemic years, a reduction in antiviral use in patients with influenza infection was observed. Among all cases of influenza in this study, only 35% of the patients received oseltamivir. Moreover, just 25% of these patients had treatment initiated within 48 hours of symptom onset, although all symptomatic patients in this period of disease should be treated. The Brazilian Health Ministry recommends treatment initiation in all patients within <48 hours of symptom onset and in those with SARI diagnosis regardless of the timing of symptoms^5^.

Influenza-infected patients showed more cases of fever (93%), cough (100%), and myalgia than those in patients infected by ORVs (Table 2). Even though influenza infections comprised only 10% of the total cases of RV detected in this study, these viruses represented 44% of all cases with myalgia. Furthermore, influenza infections tended to cause more severe disease, with more cases of SARI; however, this finding should be confirmed by prospective studies with higher numbers of patients. Of the 775 total cases in this study, 70% (535/775) were diagnosed with SARI and 57% (444/775) were virus-positive, 8% of which were positive for influenza (Table 2). Adjusted analysis showed that patients who were older, with SARI, and with myalgia had increased chances of positive results for influenza. Therefore, older patients with SARI diagnosis should be treated as having influenza infections until the laboratory investigation is completed. 

**TABLE 2:** Comparisons of demographic and epidemiological data between hospitalized patients with influenza (N=46) and other CRV infections (N=398), 2012-2013.

	Influenza	Other CRVs	Unadjusted analysis	Adjusted analysis		
	Number positive/total	Number positive/total	***p* value**	***p* value**	OR	CI 95%
	(%)	(%)				
Virus co-infection	8/46 (17.4)	116/398 (29)	0.115^a^	-	-	-
Sex						
Male	25/46 (54)	204/398 (51)	0.89^a^	-	-	-
Age, years			**<0.0** ^b^		-	-
<2	16/46 (35)	290/398 (73)	-	-	-	-
2 to 5	6/46 (13)	35/398 (9)	-	-	-	-
5 to 14	3/46 (7)	22/398 (6)	-	-	-	-
14 to 60	17/46 (37)	39/398 (9)	-	-	-	-
>60	4/46 (8)	12/398 (3)	-	-	-	-
Median age (IQR 25-75)	5.4 (0.6-42)	0.7 (0.2-2.3)	**<0.01** ^c^	**0.003**	**1.022**	**1.007-1.037**
Nosocomial Infection	2/46 (4)	51/398 (13)	0.145^b^	-	-	-
Time of hospitalization, days						
Median, (IQR 25-75)	8.5 (6-13.2)	7 (4-16)	0.18^c^	-	-	-
Influenza Vaccine (<1 year)	12/46 (26)	-	NA	-	-	-
Use of oseltamivir	16/46 (35)	-	NA	-	-	-
Clinical findings						
Fever	43 (93)	311 (78)	<0.01^b^	0.42	-	-
Cough	46 (100)	353 (89)	0.08^b^	0.46	-	-
Dyspnea	43 (93)	347 (87)	0.33^b^	-	-	-
Myalgia	11 (24)	14 (4)	**<0.01** ^b^	**<0.01**	**5.582**	**2.187-13.898**
Comorbidities						
None	26 (56)	266 (66)	0.21^a^	-	-	-
Immunosuppression	10/26 (38)	62/132 (47)	0.35^a^	-	-	-
Chronic lung disease	11/26 (42)	53/132 (40)	0.07^a^	-	-	-
Radiographic findings						
Missing value	13 (28)	188 (47)	-	-	-	-
Normal	2/33 (6)	37/210 (17)	0.12^b^	-	-	-
Interstitial infiltrate	12/33 (36)	68/210 (32)	0.69^b^	-	-	-
Pulmonary consolidation	9/33 (25)	57/210 (27)	1.00^b^	-	-	-
Mixed pattern	9/33 (25)	12/210 (5)	**0.04** ^b^	0.81	-	-
Mechanical ventilation	13 (28)	82 (21)	0.31^a^	-	-	-
ICU stay	18 (39)	129(32)	0.45^a^	-	-	-
Died	4 (9)	22 (6)	0.33^b^	-	-	-
Diagnosis of SARI	41 (89)	298 (75)	**0.04** ^a^	**0.05**	-	-

**In bold:** statistically significant; **CRV:** community-acquired respiratory virus; **IQR:** interquartile range; **ICU:** intensive care unit; **ARD:** acute respiratory distress; **NA:** not applicable; **OR:** odds ratio. ^a^Chi-squared test. ^b^Fisher’s exact test. ^c^Mann-Whitney U test.

Chest X-ray was performed in 55% (201/444) of the positive patients, 80% of which showed some abnormality. Significant alterations such as interstitial pneumonia (36% and 32%, *p*=0.69), pulmonary consolidation (25% and 27%, *p*=1.00) and mixed patterns (25% and 5%, *p*<0.05) were reported in patients infected with influenza and ORVs, respectively. Most studies neglected the analysis of X-ray patterns in respiratory tract infections caused by influenza, which showed statistical relevance in this analysis. In 18 influenza-infected patients, the radiographic findings were either consolidation or mixed patterns, none of which had simultaneous bacterial detection. These results emphasize that severe tissue damage is found in hospitalized patients with pneumonia caused by influenza.

Comparison of the epidemiological and clinical characteristics reported in this study between patients with influenza virus A and B infections revealed no statistically significant results differences, although the low prevalence of influenza B virus may have impaired this assessment. 

Studies that aimed to compare the clinical presentation of influenza patients across virus types and subtypes/lineages have reported divergent results; however, in general, despite differences in age distribution, the clinical illnesses produced by the different influenza virus types and subtypes are indistinguishable^11^. 

In both years, influenza cases were more common in June and July. The study results showed an association between influenza rates and decreased temperature (Figure 2). However, unlike previous findings, no association was observed between influenza circulation and rainfall during the study period^12^. Studies on influenza seasonality in Brazil have shown distinct patterns of viral circulation; in the Northeast region, influenza circulates in the first 4 months of the year, overlapping with a period of higher humidity in that area^13^ ^,^ ^14^. These different circulation profiles due to the climatic conditions of each region impact the vaccine effectiveness, which in tropical regions has been carried out in a period subsequent to viral circulation. 

**FIGURE 2:**
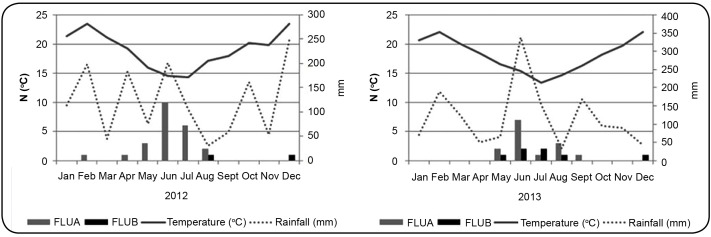
Influenza infection seasonality and association with monthly median temperature and rainfall, 2012 and 2013.

The 10.4% influenza positivity among samples found is similar to that in previous reports^7^ ^,^ ^12^ ^,^ ^14^; however, the present study tested a larger number of influenza A virus samples for subtypes. In Brazil, there was a non-homogeneous distribution of influenza A subtypes. Laboratory surveillance data in the Southern region showed a higher frequency of influenza A/H1N1pdm than A/H3N2, at 61% and 38%, respectively. In the Northeastern region, which has different climatic conditions, there was circulation of influenza A/H3N2 in the first 16 epidemiological weeks (EW) and then an increased detection of H1N1pdm between the 20^th.^ and 30^th^ EW, but with a lower intensity than occurred in the Southeastern and Southern regions^4^. These findings highlight the need to subtype influenza samples from distinct regions to improve the surveillance system. 

Among influenza B cases, 78% occurred in 2013 and reintroduction of the Victoria lineage was observed. In 2013, 89% of the influenza B cases in the region were antigenically categorized as the B/Brisbane/60/2008, Victoria lineage^4^. In both years, the predominant circulating lineage was Victoria-like, a variant different from that selected for vaccine composition (A/H1N1pdm and A/H3N2, a virus similar to the B/Wisconsin/1/2010, Yamagata lineage^10^), highlighting the importance of updated information on circulating viruses to determine the vaccine composition. These findings emphasize the importance of public vaccination and studies on circulating virus strains.

Although the difference was not statistically significant, the rate of severe cases among influenza B (5/9 cases, 55%) was higher than previously reported^3^. More recent reports have also shown similar findings, with incidences of severe disease in influenza B of up to 50%^15^. This finding underscores the relevance of influenza B infection, which has usually been associated with mild disease

This study had some limitations. The retrospective data collection may have contributed to the loss of some clinical and laboratory information. The parameters with higher rates of missing data were the vaccination status and the radiographic patterns. A prospective study involving a wider period of time would improve the analysis, allowing evaluation of the seasonality of influenza B cases.

In conclusion, influenza infections were associated with seasonal and severe disease, occurred more commonly in older patients, and usually, in a non-immunized population. The influenza treatment rates should be increased and treatment should be initiated earlier, especially in critically ill patients. An increased frequency of influenza B infections occurred in 2013, probably due to a mismatch between the vaccine and the circulating lineage. Strengthening surveillance systems within institutions is important to rapidly identify the circulation of pathogens that present a risk to public health.
